# Other PHAC publications

**DOI:** 10.24095/hpcdp.46.1.06

**Published:** 2026-01

**Researchers from the Public Health Agency of Canada also contribute to work published in other journals and books. Look for the following articles published in 2025:**

Andreacchi AT, Fuller AE, Smith PM, **Blair A**, Harris A, Carnide N, et al. Employment quality and suicide, drug poisoning, and alcohol-attributable mortality. Am J Epidemiol. 2025;194(8):2164-73. https://doi.org/10.1093/aje/kwaf018

Chateauvert J, Dufort P, Folco JD, Gunter C, Maki K, **Morales Hudon AM**, et al. Social innovation for real-world transformation: roadmaps for changing the world. Bristol (UK): Bristol University Press; 2025. 204 p.

Lennord E, Amoako E, Rajasingham M, Kirubarajan A, D’Souza R, Malham I, **Dzakpasu S**, et al. Maternal and neonatal health in Canada’s Black communities: a scoping review of epidemiologic studies. Can J Public Health. 2025. https://doi.org/10.17269/s41997-025-01102-9

Malham I, Seymour RJ, Ashraf R, Gehrke P, Beyene J, Seedu T, […] **Dzakpasu S**, et al. Feasibility of establishing a Canadian Obstetric Survey System (CanOSS) for severe maternal morbidity: results of a nationwide survey. Public Health Pract. 2025;10:100650. https://doi.org/10.1016/j.puhip.2025.100650

**Prince SA, Enns A, Lang JJ, Lancione S, de Groh M, Geneau R**. Built environment and physical activity evidence gaps: a content analysis of published systematic reviews. Int J Community Wellbeing. 2025;8(3):797-831. https://doi.org/10.1007/s42413-025-00263-2

Thombs BD, **Traversy G**, Reynolds DL, Lang E, Groulx S, Wilson BJ; Canadian Task Force on Preventive Health Care. Recommendations on interventions for tobacco smoking cessation in adults in Canada. CMAJ. 2025;197(28):E846-61. https://doi.org/10.1503/cmaj.241584

Villeneuve PJ, Cottagiri SA, **Jiang Y, de Groh M**, Fuller-Thomson E. Residential greenness and reduced depression during COVID-19: longitudinal evidence from the Canadian Longitudinal Study on Aging. PLoS One. 2025;20(8):e0329141. https://doi.org/10.1371/journal.pone.0329141

Ward B, Edgar NE, Ahluwalia C, Huang E, Corsi D, Cameron DW, […] **Orpana H**, et al. Mental health and cognitive outcomes in patients six months after testing positive compared with matched patients testing negative for COVID-19 in a non-hospitalized sample: a matched retrospective cohort study. Int J Environ Res Public Health. 2025;22(8):1249. https://doi.org/10.3390/ijerph22081249

